# Investigating the Functional Role of Hypothetical Proteins From an Antarctic Bacterium *Pseudomonas* sp. Lz4W: Emphasis on Identifying Proteins Involved in Cold Adaptation

**DOI:** 10.3389/fgene.2022.825269

**Published:** 2022-03-11

**Authors:** Johny Ijaq, Deepika Chandra, Malay Kumar Ray, M. V. Jagannadham

**Affiliations:** ^1^ Metabolomics Facility, School of Life Sciences, University of Hyderabad, Hyderabad, India; ^2^ CSIR-Centre for Cellular and Molecular Biology, Hyderabad, India

**Keywords:** *Pseudomonas* sp. Lz4W, LC-MS/MS, hypothetical proteins, cold adaptation, functional annotation

## Abstract

Exploring the molecular mechanisms behind bacterial adaptation to extreme temperatures has potential biotechnological applications. In the present study, *Pseudomonas* sp. Lz4W, a Gram-negative psychrophilic bacterium adapted to survive in Antarctica, was selected to decipher the molecular mechanism underlying the cold adaptation. Proteome analysis of the isolates grown at 4°C was performed to identify the proteins and pathways that are responsible for the adaptation. However, many proteins from the expressed proteome were found to be hypothetical proteins (HPs), whose function is unknown. Investigating the functional roles of these proteins may provide additional information in the biological understanding of the bacterial cold adaptation. Thus, our study aimed to assign functions to these HPs and understand their role at the molecular level. We used a structured *insilico* workflow combining different bioinformatics tools and databases for functional annotation. *Pseudomonas* sp. Lz4W genome (CP017432, version 1) contains 4493 genes and 4412 coding sequences (CDS), of which 743 CDS were annotated as HPs. Of these, from the proteome analysis, 61 HPs were found to be expressed consistently at the protein level. The amino acid sequences of these 61 HPs were submitted to our workflow and we could successfully assign a function to 18 HPs. Most of these proteins were predicted to be involved in biological mechanisms of cold adaptations such as peptidoglycan metabolism, cell wall organization, ATP hydrolysis, outer membrane fluidity, catalysis, and others. This study provided a better understanding of the functional significance of HPs in cold adaptation of *Pseudomonas* sp. Lz4W. Our approach emphasizes the importance of addressing the “hypothetical protein problem” for a thorough understanding of mechanisms at the cellular level, as well as, provided the assessment of integrating proteomics methods with various annotation and curation approaches to characterize hypothetical or uncharacterized protein data. The MS proteomics data generated from this study has been deposited to the ProteomeXchange through PRIDE with the dataset identifier–PXD029741.

## Introduction

Bacterial adaptation to cold environments is a fascinating area of research and reveals a great potential for biotechnological applications. The adaptive capability of bacteria to low temperatures depends on regulating many integrated signalling and molecular mechanisms which enable bacteria to sense the environmental changes, process the signals, and orchestrate the physiological changes accordingly (Moradali et al., 2017). Studies on cold adaptation of Antarctic bacteria revealed many of their survival strategies like the ability to control membrane fluidity, catalyze biochemical reactions at frozen temperatures, sense the temperature cues, regulate transcription and translation machinery, and modify protein conformation (Ray et al., 1994; Chintalapati, et al., 2004; Shivaji and Prakash, 2010; Prakash et al., 2010). Modifications in the cell envelop components such as peptidoglycan, lipopolysaccharides (LPS), and exopolysaccharides appear to be an important strategy for cold adaptation (Tribelli and Lopez, 2018). Additionally, cold adaptation in psychrophiles is attributed to membrane proteins, chaperones, RNA helicases, cold shock proteins, carotenoids, short- and branched-chain fatty acids, and antioxidative enzymes. Furthermore, an increase in the expression of proteins related to replication, transport, and folding was reported as a mechanism of cold adaptation (De Maayer et al., 2014; Bargiela et al., 2020). Likewise, changes in the catalytic activity of the enzymes, changes in central metabolic activities, post-translational modifications, synthesis of storage polymers, like polyhydroxyalkanoates (PHAs) were reported to be advantageous for bacterial adaptation to cold temperatures (Tribelli and Lopez, 2018; Bargiela et al., 2020).

In our laboratory, we have been using *Pseudomonas* sp. Lz4W as a model system to understand the cold adaptation of bacteria (Ray et al., 1998). *Pseudomonas* sp. Lz4W is a gram-negative bacterium belonging to the γ-proteobacteria class. This strain was isolated from the Schirmacher Oasis, Antarctica (70°45′12″S and 11°46′E) (Shivaji et al., 1989). It is capable of surviving zero and sub-zero temperatures. Under laboratory conditions, it was observed to be capable of growing up to 30°C, whereas 22°C being the optimum growth temperature (Kannan et al., 1998). Pandiyan and MK Ray reported the first draft genome sequence of this bacterium to decipher the genomic basis of its psychrophilicity (Pandiyan and Ray, 2013). Over the past decade, extensive research studies have focused at deciphering the underlying molecular mechanisms of *Pseudomonas* sp. Lz4W adaptability to cold temperatures (Chattopadhyay and Jagannadham 2001; Kumar et al., 2002; Jagannadham and Chowdhury, 2012).

The genetic architecture of an organism determines the ability of an organism to respond to environmental variations. To address this, the genome and the proteome of *Pseudomonas* sp. Lz4W has been investigated by previous studies. However, there are few coding sequences in the genome that are predicted to code for proteins but having no evidence of expression at the translation level. Proteins encoded by such sequences were assigned as “hypothetical” or “uncharacterized” (Desler et al., 2009; Dall'Agnol et al., 2014). These hypothetical proteins were found to constitute a major portion of microbial genomes. From recent studies, it was evident that functional characterization of hypothetical proteins (HPs) could provide additional understanding of the physiological architecture of the cell (Rabbi et al., 2021; Araújo et al., 2020; Pranavathiyani et al., 2020; Sen and Verma, 2020; da Costa et al., 2018; Naqvi et al., 2015; Shahbaaz et al., 2013). Genomic analysis of *Pseudomonas* sp. Lz4W also predicted few ORFs as hypothetical proteins. Our previous proteomic studies on membrane proteins of *Pseudomonas* sp. Lz4W also revealed the expression of a few HPs (Jagannadham et al., 2011; Jagannadham and Chowdhury, 2012). But functional studies on these hypothetical proteins are lacking. Hence, studying these functionally unknown sequences could provide additional insight into potential mechanisms governing cold adaptation of *Pseudomonas* sp. Lz4W.

HPs were found to be important in connecting the missing links in genomic and proteomic information. From our previous study (Ijaq et al., 2020) using mass spectrometry (MS)-based proteomics, we provided evidence of expression for HPs at the protein level. Furthermore, these days a wide range of computational biology tools are available to unravel the functional roles of the identified hypothetical protein sequences (Ijaq et al., 2015; Ijaq et al., 2019). Keeping this in mind, for this study, we set an objective to identify and characterize the hypothetical sequences from the genome of *Pseudomonas* sp. Lz4W. Proteomics-based methods combined with annotation-based workflow were used to accomplish the functional characterization of HPs concerning their role in cold adaptation. This annotation helped in assigning various classes to the HPs such as membrane proteins, lipoproteins, transporters, enzymes, chaperones and virulence factors. We believe that this study will give insights regarding the functional roles of HPs present in *Pseudomonas* sp. Lz4W. Further experimental validation of these identified proteins may eventually help in developing new biotechnological applications.

## Materials and Methods

### Bacterial Culture and Growth Conditions

*Pseudomonas sp*. Lz4W was isolated from the Schirmacher Oasis region of Antarctica (70°45′12″ S and 11°46′ E) (Shivaji et al., 1989). The strain was cultured using Antarctic bacterium media (ABM) comprising of peptone 5%, yeast extract 2%, and agar 1.5% (for solid media). Bacterial cells were grown in three different batches at 4°C and the cells were harvested in the early log phase. The bacterial cultures were then centrifuged (8000 rpm for 20 min) and the cell pellets were further processed to prepare the sample repeats for proteomic analysis.

### Protein Isolation

Cell pellets were resuspended in lysis buffer (20 mM Tris-Cl pH 8, 150 mM NaCl, and 2 mM EDTA). PMSF (Phenylmethylsulfonyl fluoride) and protease inhibitor cocktail were added to the suspension. Resuspended cells were then lysed with 30 min lysozyme treatment at 4°C. Cell lysis was furthered by sonication (3-5 cycles of 30 s ON and 30 s OFF, at a medium frequency) using Diagenode’s Bioruptor Plus sonication system. Cell debris and unbroken cells were removed as pellets by centrifugation at 10,000 rpm at 4°C for 10 min. The supernatant was collected and this constitutes the whole-cell extract. This whole-cell extract was treated with dithiothreitol (1 mM) and iodoacetamide (Gundry et al., 2009) to reduce the disulfide bonds of proteins. The supernatant was then further fractionated by ultracentrifugation (100,000Xg for 1 h) to identify the low abundant proteins. This step resulted in two fractions–supernatant as soluble protein fraction of whole-cell extract and pellet as an insoluble fraction (Ames, 1974).

Proteins present in the soluble fraction were precipitated following the acetone precipitation method (Nejadi et al., 2014) with small modifications. The soluble fraction was mixed with chilled acetone in a 1:3 ratio and kept overnight at −20°C. Proteins precipitated were then separated as pellet using centrifugation (13,000 rpm at 4°C for 20 min). Pellet was air-dried and subsequently resuspended in 1X Laemmli buffer. Insoluble fraction pellet obtained from the previous step was also re-suspended in 1X Laemmli buffer.

### SDS PAGE and In-Gel Digestion

Soluble and insoluble fractions were collected in equal amounts (40 µg each) and subjected to SDS-PAGE in two different lanes. The gel was stained with Coomassie Blue. A small portion of gel with prominent bands was excised and used for in-gel digestion with trypsin as described earlier with slight modifications (Jagannadham 2008). Extracted peptides were desalted by using C18 resin-based Zip-Tips obtained from Millipore.

### Mass Spectrometry Analysis and Data Acquisition

LC-MS/MS analysis of tryptic digested peptides was carried out using Q Exactive HF (Thermo Fisher Scientific). Digested peptides were reconstituted in 0.1% formic acid and separated by nano-LC using an Easy-nLC 1,200 (Thermo Fisher Scientific, United States). An equal amount of peptide samples (400 ng each) were loaded into NanoDrop 2000/2000c spectrometer (Thermo Fisher Scientific) with absorbance at 205 nm for quantification. PepMap™ RSLC C18 column (75 µm × 15 cm length, 3 µm particle size, and 100 A^0^ pore size, Thermo Fisher Scientific, United States) was used to separate the tryptic digested peptides. A binary solvent system was used with the mobile phases comprising (A) 5% acetonitrile with 0.1% (vol/vol) formic acid and (B) 95% acetonitrile with 0.1% (vol/vol) formic acid. Peptides were eluted with a linear gradient of Buffer A to Buffer B with a flow rate of 300 nL/min over a 90 min gradient run. Separations are performed by changing the percentage of B. The binary gradient elution was as follows: 0 min, 0% B; 79 min, 90% B; 90 min, 0% B. For MS scan, mass range *m/z* 400–1750 was used. Dynamic exclusion interval of 15 s was selected with exclude isotope mode ON. For MS/MS, normalized collision energy of 28% was used for collision-induced dissociation (CID) activation. To retrieve MS/MS spectra, top 10 peaks was selected as scan event.

### Protein Identification

Raw files generated by Q Exactive HF were submitted to proteome discoverer software (version 1.4, Thermo Fischer). SEQUEST search engine was used to search the mass spectra of peptides against the sequence database. For the identification of the proteins, all MS/MS spectra of peptides were searched against *Pseudomonas* sp. Lz4W database (GenBank Accession: CP017432.1, BioProject: PRJNA170013) following the criteria: precursor mass tolerance/peptide tolerance, ± 10 ppm; fragment mass tolerance/MS/MS tolerance, ± 0.6 Da; spectra selection range of precursor mass, 350–5,000 Da; dynamic modification, oxidation of methionine (15.99 Da); and static modification, carbamidomethylation of cysteine (57.02 Da). Two missed cleavages were set for the identification of proteins. False discovery rate (FDR) was set to 1% for the identification of peptides with high confidence.

Proteome profiling of triplicate cultures resulted in three sets of protein data - Set A, Set B, and Set C, identifying 949, 919, and 872 proteins respectively from each set. Initially, we searched for the common proteins across the three sets. From the common proteins, proteins annotated as ‘hypothetical proteins’ were retrieved and further investigated for their functional roles.

### Sequence Retrieval

*Pseudomonas sp*. Lz4W genome was used for retrieving the sequences. *Pseudomonas sp*. Lz4W genome was sequenced and assembled in our laboratory (Pandiyan and Ray, 2013). Its genome consists of 5,018,820 bp corresponding to 4493 genes, of which 4,393 genes code for the proteins (coding sequences). From these, a total of 743 coding sequences (CDS) were annotated as hypothetical proteins. Genomic data was retrieved from the National Center for Biotechnology Information database (https://www.ncbi.nlm.nih.gov/nuccore/) under accession number CP017432. The sequences of the expressed HPs were retrieved in FASTA format from NCBI (https://www.ncbi.nlm.nih.gov/) using their primary accession numbers. Corresponding UniProt entries were crosschecked in the Uniprot database (UniProt Proteome ID: UP000011925) to retrieve the UniProt accession numbers.

### Functional Annotation

**FIGURE 1:**
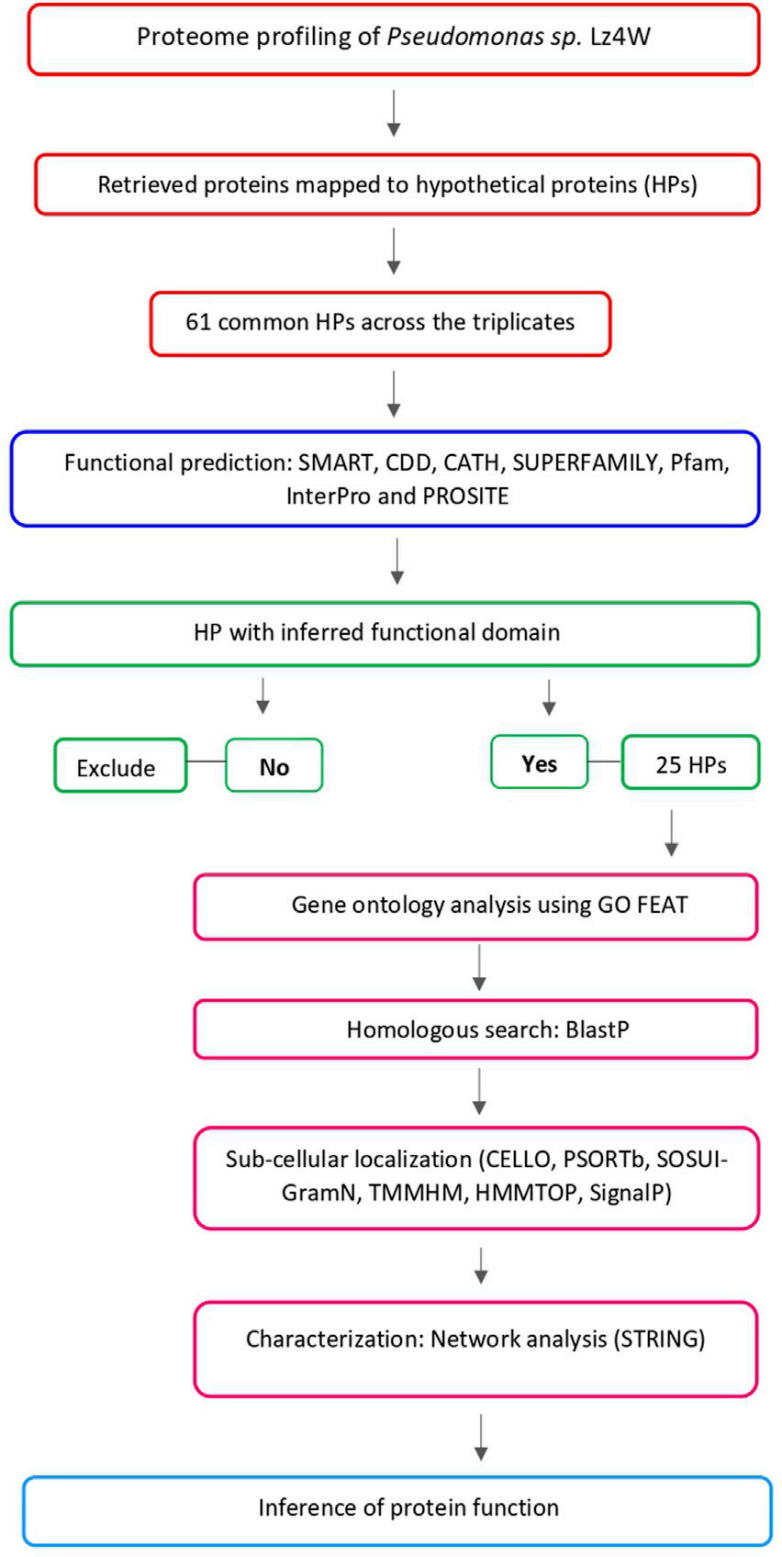
Workflow used for the annotation of the identified hypothetical proteins.

Hypothetical proteins that were identified at the translation level were subjected to functional annotation to uncover their functional roles. An overview of the annotation workflow is presented in Figure 1. First, they were provided with putative functional annotation based on the functional domains and motifs using various publicly available bioinformatics tools. Simple Modular Architecture Research Tool (SMART) (version 9.0) was used to predict the function of a protein based on the domain architecture. It searches proteome information from Swiss-Prot, SP-TrEMBL, and stable Ensembl for a similar domain (Letunic et al., 2012). CATH (version 4.3) (Sillitoe et al., 2015) was used to classify the domains within the structural hierarchy. Conserved Domain Database (CDD) (version 3.19) (Lu et al., 2020) was used to explore conserved domains that are associated with molecular function. Pfam (version 35.0) (Finn et al., 2014), SUPERFAMILY (version 2.0) (Gough et al., 2001), InterPro (version 87.0) (Blum, et al., 2021), and PROSITE (release 2021_04) (Sigrist et al., 2013) were used to classify the HPs into functional families to predict the function based on similarity. InterProScan (version 5.45–87.0) was used as the underlying software to search the hypothetical protein sequences against InterPro’s protein family signatures. (Blum, et al., 2021). Functional domain was inferred to each hypothetical protein if the similar domain was found consistently across the databases used. For all the databases, we used default parameters.

Functional annotations were further strengthened by matching the protein functional domains, motifs, and families along with GO terms. GO FEAT tool was used for GO based functional annotation of HP sequences (Araujo et al., 2018). E-value of 1e-03 was selected to ensure significant matches. Subsequently, sequence similarity search was performed to identify if a hypothetical protein sequence is homologous to sequences that are already available in existing databases. BlastP (Altschul et al., 1990; Altschul et al., 1997) was used against NCBI nonredundant (nr) database, and hits with an identity >90% were considered.

### Sub-Cellular Localization

Knowledge about the subcellular localization of a protein can provide an estimate of a protein’s function at the cellular level. PSORTb, CELLO and SOSUI-GramN were used to assign the sub-cellular location of the HPs. PSORTb (version 3.0.3) provides prediction of subcellular localization of bacterial and archaeal proteins. (Gardy et al., 2003). CELLO (version 2.5) is a two level support vector machine (SVM) based system used for the prediction of localization of proteins from both bacteria and eukaryotes. (Yu et al., 2006). SOSUI-GramN system is specific to predict the extracellular and membrane protein localization in gram negative bacteria. (Imai et al., 2008). SignalP (version 5.0) was used to predict the presence of signal peptide cleavage sites (Petersen et al., 2011). TMHMM (version 2.0) (Krogh et al., 2001) and HMMTOP (version 2.0) (Tusnády and Simon, 2001) was used to predict the transmembrane helices in proteins thus identifying the membrane proteins.

### Protein-Protein Interaction Analysis

STRING (version 11.5) was used for protein-protein interaction analysis of the HPs. It provides functional associations between proteins based on the information from different sources like scientific literature, experimental evidence, predictions from co-expression, genome context, curated pathway databases and calculates the final ‘confidence score’ for each association (Szklarczyk et al., 2021). For this study, a confidence score above 0.7 was considered to ensure the reliability of the association. *Pseudomonas sp*. Lz4W reference genome was used to generate the interaction networks. Interactions were further visualized using Cytoscape (version 3.9.0) (Shannon et al., 2003). All the bioinformatics tools and databases used in this study are listed in Supplementary Table S1.

## Results and Discussion

**FIGURE 2:**
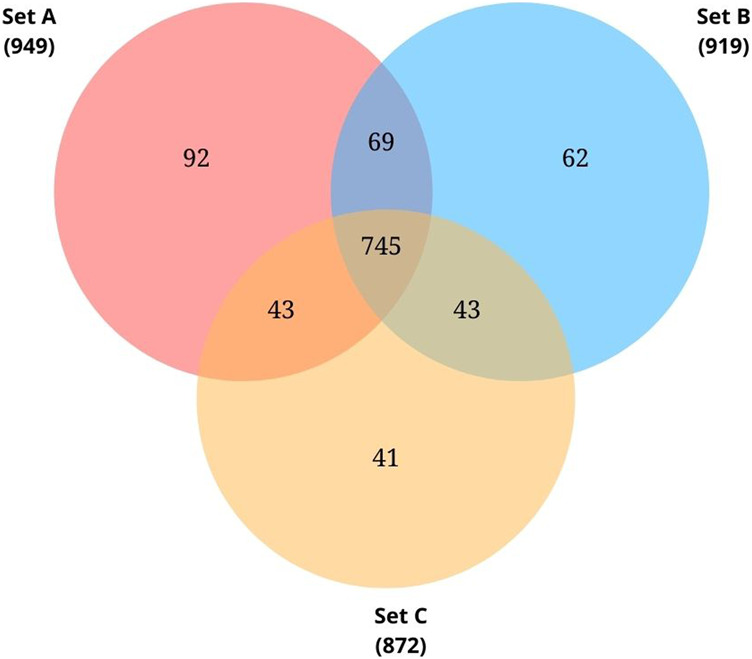
Venn diagram showing common proteins identified from the biological triplicates using LC-MS/MS analysis. Total proteins identified from each set: 949 from Set A, 919 from Set B, 872 from Set C. Of these 745 proteins were found to be common.

The complete genome sequence for the species *Pseudomonas* sp. Lz4W was deposited in the NCBI database in 2013 by Ray and colleagues (Pandiyan and Ray, 2013). As on November 2021, a total of 4393 protein-coding genes were predicted on this genome. Among these, 743 CDS (16.9%) were labelled as hypothetical proteins whose function is unknown. In the present study, we tried investigating the protein expression of *Pseudomonas* sp. Lz4W during cold adaptation to check whether any of these CDS labelled as HPs are expressed at the protein level. Proteome profiling of the triplicate cultures resulted in 949, 919, and 872 proteins respectively **(** Supplementary Table S2). 745 common proteins were identified across the three sets. From these 745 proteins, 61 proteins were found to be annotated as “hypothetical proteins” **(** Figure 2). A complete list of these 61 HPs and their MS data is presented in Supplementary Table S3 **.** Uniprot annotation of the corresponding entries is shown in Supplementary Table S4. From the UniProt information, it was found that all these 61 HPs are either predicted proteins or proteins inferred from homology. From this proteomic analysis, identifying these proteins at the translational level provides evidence for their expression in the tested growth conditions. The sequences of these proteins were extracted from NCBI and used for further functional characterization using available bioinformatics tools and resources.

### Functional Annotation Using Domain Analysis

Initially, we looked for the functional domains in the sequences. Domains are functional and structural units of proteins that are evolutionarily conserved across each protein family or superfamily (Ponting and Russell, 2002). As they are highly conserved within a functional protein family, looking for a presence of a functional domain in the hypothetical protein sequences could provide insights into their function (Eisenstein et al., 2000; Sen and Verma, 2020). Sequences of all the 61 HPs were extensively analyzed for the presence of functional domains using SMART, CDD (CD-Search tool), CATH, Pfam, SUPERFAMILY, InterPro, and PROSITE. The predicted results were shown in Supplementary Table S5. Out of 61 HPs, 25 proteins were assigned with functional domains. Putative function was assigned to these proteins only if a similar functional domain is identified across the different tools and databases used. For the remaining 36 proteins, no specific conserved domains were found for 20 proteins and the remaining 16 proteins were found to have a DUF (domain of unknown function) domain, whose function is not yet characterized (Table 1).

**TABLE 1:** Hypothetical proteins identified from Pseudomonas sp. Lz4W along with their functional analysis.

S.No	GenBank accession	UniProt ID	Putative function	Category
Proteins assigned with functional domains				
1	AUB73822.1	A0A2H4VVI5	Endolytic peptidoglycan transglycosylase (RlpA)	Enzymes
2	AUB74731.1	A0A2H4VY66	Thiosulfate sulfurtransferase	
3	AUB74781.1	A0A2H4VYB4	ATPase activity	
4	AUB74810.1	A0A2H4VYB1	Pyrimidine/purine nucleoside phosphorylase	
5	AUB77590.1	A0A2H4W6A7	Glycosyl transferases group 1	
6	AUB75791.1	A0A2H4W154	Ligase, Condensation domain	
7	AUB75792.1	A0A2H4W178	Ligase, Condensation domain	
8	AUB75991.1	A0A2H4W1Q4	Sulfatase	
9	AUB76494.1	A0A2H4W376	Flavinator of succinate dehydrogenase	
10	AUB76830.1	A0A2H4W456	Aminoglycoside phosphotransferase (APH)	
11	AUB74223.1	A0A2H4VWR7	Outer membrane protein	Membrane proteins
12	AUB76580.1	A0A2H4W3E2	Outer membrane protein (OmpA)	
13	AUB76959.1	A0A2H4W4H7	Outer membrane protein (OmpA)	
14	AUB77315.1	A0A2H4W5I1	Outer membrane porin (OprD)	
15	AUB73831.1	A0A2H4VVK8	Lipopolysaccharide-assembly lipoprotein (LptE)	Lipoproteins
16	AUB74026.1	A0A2H4VW39	Lipoprotein	
17	AUB74624.1	A0A2H4VXV2	Lipoprotein with Alanine-zipper	
18	AUB75187.1	A0A2H4VZE1	Intermembrane phospholipid transport system lipoprotein (MlaA)	Transport proteins
19	AUB75954.1	A0A2H4W1N5	Copper resistance protein C (CopC), Copper transport protein	
20	AUB77446.1	A0A2H4W5Z0	Tim 44 like domain	
21	AUB76897.1	A0A2H4W4C7	HSP70 family of molecular chaperone	Chaperones
22	AUB76544.1	A0A2H4W3C6	Outer membrane chaperone (skp)	
23	AUB73565.1	A0A2H4VUU7	YceI-like domain	Cellular process
24	AUB73322.1	A0A2H4VU41	Virulence factor	Virulence factors
25	AUB74265.1	A0A2H4VWT9	Putative quorum-sensing-regulated virulence factor	
Proteins assigned with domain of unknown function				
26	AUB73312.1	A0A2H4VU37	Domain of unknown function	Domain of Unknown Function (DUF)
27	AUB73583.1	A0A2H4VUV2	Domain of unknown function	
28	AUB73639.1	A0A2H4VV22	Domain of unknown function	
29	AUB73986.1	A0A2H4VW02	Domain of unknown function	
30	AUB74271.1	A0A2H4VWW5	Domain of unknown function	
31	AUB74729.1	A0A2H4VY42	Domain of unknown function	
32	AUB74842.1	A0A2H4VYJ4	Domain of unknown function	
33	AUB77556.1	A0A2H4W6A6	Domain of unknown function	
34	AUB75101.1	A0A2H4VZ73	Domain of unknown function	
35	AUB75536.1	A0A2H4W0E2	Domain of unknown function	
36	AUB75846.1	A0A2H4W1A8	Domain of unknown function	
37	AUB76324.1	A0A2H4W2Q7	Domain of unknown function	
38	AUB76750.1	A0A2H4W3Y2	Domain of unknown function	
39	AUB77015.1	A0A2H4W4N4	Domain of unknown function	
40	AUB77395.1	A0A2H4W5R3	Domain of unknown function	
41	AUB75938.1	A0A2H4W1Q3	Domain of unknown function	
Proteins identified with no functional domain				
42	AUB73328.1	A0A2H4VU50	None	Uncharacterized
43	AUB73856.1	A0A2H4VVM1	None	
44	AUB73929.1	A0A2H4VVW4	None	
45	AUB73991.1	A0A2H4VW15	None	
46	AUB73994.1	A0A2H4VW21	None	
47	AUB74225.1	A0A2H4VWQ3	None	
48	AUB74336.1	A0A2H4VWZ7	None	
49	AUB74383.1	A0A2H4VX83	None	
50	AUB74509.1	A0A2H4VXH3	None	
51	AUB74552.1	A0A2H4VXN7	None	
52	AUB74606.1	A0A2H4VXS5	None	
53	AUB74636.1	A0A2H4VXV4	None	
54	AUB75651.1	A0A2H4W0S2	None	
55	AUB75652.1	A0A2H4W0U3	None	
56	AUB75757.1	A0A2H4W111	None	
57	AUB75993.1	A0A2H4W1U9	None	
58	AUB76598.1	A0A2H4W3K1	None	
59	AUB76896.1	A0A2H4W4C0	None	
60	AUB77182.1	A0A2H4W552	None	
61	AUB77347.1	A0A2H4W5N7	None	

**FIGURE 3:**
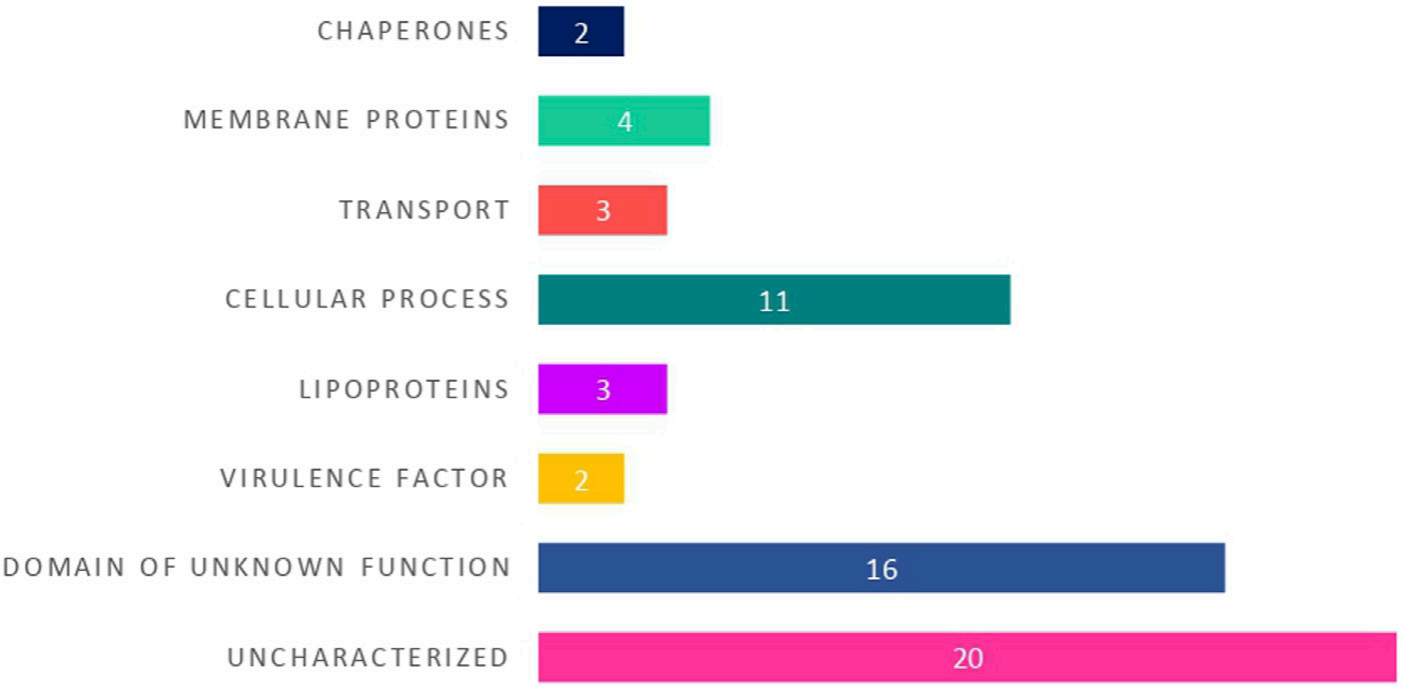
The distribution of 25 hypothetical proteins into different functional groups based on their predicted protein domains and families.

Based on the identified putative function, the 25 HPs with known functional domains were categorized into different functional groups namely membrane proteins, lipoproteins, cellular process, transport proteins, chaperones and virulence factors **(** Figure 3).

### Membrane Proteins

In our analysis, a few HPs, AUB74223.1, AUB76580.1, AUB76959.1, and AUB77315.1 were predicted to be associated with outer membrane (OM). Membrane composition, structure, and proteins play a significant role in cold adaptation. From our previous studies, it was demonstrated that the membranes and membrane components play a crucial role in the cold adaptation of bacteria (Chattopadhyay and Jagannadham 2001; Kumar et al., 2002; Jagannadham and Chowdhury, 2012). Functional genomics studies of psychrophilic bacteria have shown the increased abundance of outer membrane proteins during cold adaptation (Ting et al., 2010; Frank et al., 2011). HPs AUB74223.1, AUB76580.1 and AUB76959.1 were found to contain OmpA (outer membrane protein A) like domain. Proteins with OmpA like domain play a vital role in maintaining the outer membrane structure and stability (Wang, 2002), and may serve as a porin facilitating the transport of small solutes (Smith et al., 2007; Confer and Ayalew, 2013). Hypothetical protein (HP) AUB77315.1 was found to contain the OprD domain belonging to the outer membrane porin family. OprD is one of the major porin proteins present in the outer membrane of the genus ‘*Pseudomonas*’. Members of the OprD family are involved in the specific uptake of various small molecules ranging from amino acids to peptides, usually below 200 Da (Li et al., 2012; Chevalier et al., 2017).

### Lipoproteins

Hypothetical proteins AUB73831.1, AUB74026.1, and AUB74624.1 were annotated as lipoproteins. Bacterial lipoproteins are membrane-associated proteins with hydrophilic protein moiety attached to hydrophobic fatty acid chains. These proteins are anchored to the membrane with their N-terminally linked fatty acids (Wilson and Bernstein, 2016). They are involved in diverse functions like outer membrane biogenesis, transport of various molecules, signal transduction, cell motility, and maintenance of cell shape (Narita and Tokuda, 2017). Previous studies on bacterial cold adaptation (Ting et al., 2010) have described the role of lipoproteins in optimizing membrane structure and function at low temperatures, indicating their role in cold adaptation. HP AUB73831.1 was annotated as LptE (lipopolysaccharide-assembly lipoprotein). LptE, together with LptD, assists in the proper assembly of lipopolysaccharide (Chimalakonda et al., 2011). Being a part of the cell envelope, proper assembly of LPS confers an advantage to bacterial cold adaptation (Tribelli and López, 2018). HP AUB74624.1 was predicted to be a lipoprotein with alanine-zipper.

### Cellular Process

Microbial adaptation to cold temperatures necessitates changes in metabolism which is in turn regulated by enzymes. From our analysis, 10 HPs were characterized as enzymes. HP AUB73822.1 was predicted to be Rare lipoprotein A (RlpA). RlpA is a lytic peptidoglycan transglycosylase, which catalyzes specific cleavages in peptidoglycan chains, thus regulating the biosynthesis of peptidoglycan (Jorgenson et al., 2014). Two putative ligases (AUB75791.1, AUB75792.1) were also detected in this study. They contain a condensation domain that is known to catalyze a condensation reaction to form peptide bonds through a non-ribosomal peptide (NRP) biosynthesis mechanism (Izoré et al., 2021). Bacterial NRPs can serve as antibiotics, toxins, siderophores, pigments, cytostatics, etc. (Süssmuth and Mainz, 2017). HP AUB77590.1 was predicted to be a glycosyltransferase and is assumed to be involved in the biosynthesis of lipopolysaccharide and exopolysaccharides, important components in microbial cold adaptation (Soumya and Nampoothiri, 2021; Sen and Verma 2020; Deming and Young, 2017). Additional transferases identified were thiosulphate sulphurtransferase (AUB74731.1), and aminoglycoside phosphotransferase (APH) (AUB76830.1). Aminoglycosides inhibit bacterial cell growth by impairing protein synthesis (Cavallo and Martinetto, 1981). APH inactivates aminoglycoside antibiotics via phosphorylation thereby defending the protein synthesis mechanism (Kotra et al., 2000). HP AUB76494.1 was identified to be a flavinator of succinate dehydrogenase (SdhE) (also known as Sdh5 in humans). SdhE is necessary for succinate dehydrogenase (SDH) activity which regulates cellular respiration via electron transport system. (McNeil et al., 2012). The ability to regulate cellular respiration at low temperatures could provide a distinct advantage for bacteria to cope with frozen conditions (Amato and Christner, 2009). Few other important enzymes predicted in this group of HPs were DNA polymerase III subunit with ATPase activity (AUB74781.1), sulphatase (AUB75991.1), and Pyrimidine/purine nucleoside phosphorylase (AUB74810.1).

HP AUB73565.1 was found to contain a YceI-like domain. The family of proteins with this domain was predicted to aid in isoprenoid quinone biosynthesis, thus playing a role in the electron transport system. In *E. coli*, YceI is induced by high pH (Stancik et al., 2002; Handa et al., 2005).

### Transport proteins

Regulating the proteins of the transport systems is one of the mechanisms by which the bacteria survive extreme temperatures (Baraúna et al., 2017). HP AUB75187.1 was predicted to be MlaA lipoprotein. This protein is a part of an ABC transport system that facilitates the transport of phospholipids from the outer membrane to the inner membrane which is responsible for maintaining the lipid asymmetry in the outer membrane. Such asymmetric distribution of lipids in the OM maintains the membrane structure and confers resistance to gram-negative bacteria (Malinverni and Silhavy, 2009). HP AUB75954.1 was predicted to be a copper resistance protein C (CopC), which mediates copper homeostasis (Cha and Cooksey, 1991). HP AUB77446.1 was predicted to contain a Tim44-like domain. In eukaryotes, Tim44 is a part of translocation machinery that imports nuclear-encoded proteins into the mitochondria. The bacterial homolog of Timm44, TimA, is likely to serve as a protein transporter (Clements et al., 2009).

### Chaperones

Cold injury can cause protein denaturation or misfolding. Molecular chaperones could assist in the correct folding and stability of the proteins during various stress conditions. They also help in the refolding of misfolded proteins and solubilization of the aggregated proteins. (Chattopadhyay, 2006). In this study, two proteins (AUB76544.1, AUB76897.1) were categorized as chaperones. HP AUB76544.1 was annotated as Skp. This protein (also known as OmpH and HlpA) is a molecular chaperone that is important for the proper folding and insertion of several outer membrane proteins (Walton and Sousa, 2004). HP AUB76897 was found to comprise Hsp70 like domain implying its role as a chaperone in maintaining the structural integrity and function of proteins.

### Virulence Factors

The proteins AUB73322.1, AUB74265.1 were found to be virulence factors. Virulence factors are expressed by pathogens to promote pathogenesis. They play an important role in bacterial growth and survival (Moradali et al., 2017). HP AUB74265.1 was found to be regulated by quorum sensing. (Chevalier et al., 2017). Quorum sensing (QS) is a mechanism by which bacterial cells communicate with each other using chemical signals. This helps bacterial communities to control various biological processes essential for bacterial adaptation and survival under stress (Moradali et al., 2017). The role of virulence factors in adaptive mechanisms to cold stress is not well understood.

### Gene Ontology Analysis

**FIGURE 4:**
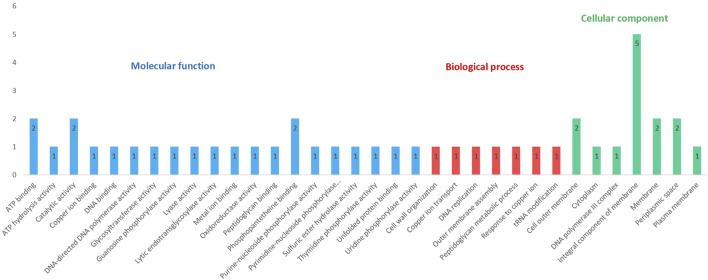
Distribution of GO terms associated with 25 hypothetical proteins among the three GO categories (Some proteins share more than one category).

These 25 proteins with known functional domains and provided putative function were further analyzed to uncover the GO terms associated with them. Out of 25 targets, 20 of them have GO term predictions. Proteins were distributed within three GO categories - molecular function, biological process, and cellular component. GO terms from three different categories are illustrated in Figure 4. Complete results from the GO FEAT platform are presented in Supplementary Table S6. 20 different GO terms were identified under molecular function. Best of these referred to ATP binding (AUB74781.1, AUB76897.1), catalytic activity (AUB75791.1, AUB75792.1), and phosphopantetheine binding (AUB75791.1; AUB75792.1). The biological process category contained 7 different GO terms, including cell wall organization (AUB73822.1), outer membrane assembly (AUB73831.1), and peptidoglycan metabolic process (AUB73822.1), processes that are central to cold adaptation. The cellular component category revealed 7 GO terms with major representation to cell membrane confirming the central role of the bacterial membrane in cold adaptation.

### Sequence Similarity Search

From BlastP analysis, 21 HPs were found to have homologous sequences in the NCBI database with described function. The remaining 4 (AUB73831.1, AUB74265.1, AUB74624.1, AUB75938.1) were matched to hypothetical proteins. The results were found to be consistent with functional domain analysis, except for HP AUB73831.1. From domain analysis, AUB73831.1 was predicted to be a Lipopolysaccharide-assembly lipoprotein (LptE). But its homologous sequences in other species of *pseudomonas* were found to be hypothetical. BlastP results are presented in Supplementary Table S7.

### Sub-Cellular Localization

Most often, protein function is correlated to its location. Therefore, the information about protein’s localization could provide supporting evidence to the functions predicted from functional domain analysis. However, emphasis was on investigating the localization of HPs that revealed functional domains. Proteins that were categorized as membrane proteins (AUB74223.1, AUB76580.1, AUB76959.1, and AUB77315.1) were predicted to be located in the outer membrane. Additionally, proteins AUB73831.1 (Lipopolysaccharide-assembly lipoprotein), AUB74624.1 (lipoprotein), and AUB77590.1 (glycosyltransferase) were also predicted to be localized in the outer membrane. Proteins that were annotated as enzymes (AUB74731.1, AUB74781.1, AUB75791.1, AUB75792.1, AUB76494.1, AUB76830.1, and AUB76897.1) were mostly found to be located in the cytoplasm. Further, proteins like AUB73565.1 (YceI family protein), AUB73822.1 (Peptidoglycan transglycosylase), AUB75954.1 (Copper resistance protein C), and AUB76544.1 (skp) were predicted to be periplasmic. Nine proteins were predicted to have transmembrane helices based on TMHMM and HMMTOP. Using SignalP- 5.0, a total of 14 proteins with cleavage sites were detected, of which 7 were “standard” secretory signal peptides (Sec/SPI) and 7 were lipoprotein signal peptides (Sec/SPII). Examination of the results reveals that, for most proteins, there was an agreement between the inferred function and the location predicted. Sub-cellular localization analysis of each HP is summarized in Supplementary Table S8 **.**

### Analysis of Protein-Protein Interactions

Further, we looked for the proteins interacting with 25 HPs. As proteins do not work alone, the function of a HP may be inferred by looking at its interaction neighbourhood. Among 25 HPs, 21 HPs were shown to have interactions with other functionally known proteins of *Pseudomonas* sp. Lz4W with a confidence score above 0.7. Out of these, annotation is not available for 3 proteins. The remaining 18 proteins were analyzed for their functions based on interactions with other proteins (Supplementary Table S9).

Three proteins AUB74223.1, AUB76580.1, and AUB76959.1, annotated as outer membrane proteins belonging to the OmpA family, have demonstrated high confidence interaction with NADH-quinone oxidoreductase subunit I (NDH-1) encoded by *nuoI*. NDH-1 has been described to be involved in shuttling electrons from NADH to quinones, as part of the bacterial respiratory chain (Leif et al., 1995). Therefore, this interaction of OmpA proteins with NDH-1 suggests their role in the electron transport system, thereby regulation of energy metabolism, which is essential for survival in frozen temperatures.

The protein AUB73831.1, assigned as LptE, revealed strong interaction with LPS-assembly protein LptD, which was shown to be involved in the assembly of lipopolysaccharide (LPS) at the surface of the outer membrane, thus confirming the function attributed to AUB73831.1 by this study (Chimalakonda et al., 2011). We confidently found protein AUB74026.1 (lipoprotein) to be interacting with penicillin-binding proteins, 1A and 1B. These penicillin-binding proteins along with other hydrolases and synthetases take part in peptidoglycan synthesis (Charpentier et al., 2002). Hence, this lipoprotein is presumed to be involved in cell wall formation by participating in the synthesis of cross-linked peptidoglycan from the lipid intermediates (Braun and Hantke, 2019).

The protein AUB73822.1, annotated as RlpA (lytic transglycosylase), exhibited high degree interaction with mrdB and mrdA proteins that are described to be associated with peptidoglycan cell wall biogenesis (Cho et al., 2016), one of the mechanisms regulated during bacterial cold adaptation. This confirms the role of the protein AUB73822.1 in peptidoglycan metabolism, as inferred by this study. Proteins AUB75791.1 and AUB75792.1 annotated as peptide synthetases presented strong interactions with 4′-phosphopantetheinyl transferase and beta-ketoacyl synthase-like protein. Studies have shown that both phosphopantetheinyl transferases (PPTs) and beta-ketoacyl synthase are components of enzymatic systems like fatty acid synthases (FAS), non-ribosomal peptide synthetases (NRPS), and polyketide synthases (PKS). They play an essential enzymatic role in fatty acid biosynthesis and synthesis of various bioactive secondary metabolites like polyketides, antibiotics, and non-ribosomal peptide metabolites (Smith et al., 2003; Copp and Neilan, 2006; Beld et al., 2014). However, the role of non-ribosomal peptides in cold adaptation is not clear. But the significance of membrane fatty acid composition for cell viability at frozen temperatures was well studied (Chintalapati et al., 2004). Protein AUB77590.1, explained as glycosyltransferase from domain analysis, exhibited interaction with a group of proteins involved in the synthesis of outer membrane LPS along with few glycosyl hydrolases that hydrolyze the glycosidic bonds between polysaccharides. This confirms the functional role of AUB77590.1 in LPS and exopolysaccharides biosynthesis, specialized adaptations to cope with cold (Soumya and Nampoothiri, 2021). The coding sequence encoding the protein AUB74731.1 with thiosulphate sulphurtransferase (TST) domain revealed a neighbourhood with genes encoding thioredoxin and ABC transporter ATP-binding protein. Sulfurtransferases play a major role in maintenance of Fe-S containing electron transport proteins. Thus, TST together with the thioredoxin system may play a role in energy transduction. In general, the thioredoxin system also functions to protect bacteria against oxidation stress. At low temperatures, oxygen solubility increases rising the levels of reactive oxygen species (ROS), leading to oxidative stress. Thioredoxin system act as scavengers of ROS, thus reducing oxidative stress (Zeller and Klug, 2006; Tribelli and Lopez, 2018). The protein AUB76830.1, with aminoglycoside phosphotransferase domain, showed interaction with a protein involved in guanine biosynthesis. But its role in cold adaptation is not known. The protein AUB76494.1, with flavinator like domain (SdhE), exhibited high confidence interaction with succinate dehydrogenase (SDH) flavoprotein subunit. This interaction confirms the role of AUB76494.1 in the flavinylation of the flavoprotein subunit of SDH, responsible for the assembly of the respiratory enzyme complex. The protein AUB74781.1 was inferred to be a DNA polymerase III subunit with ATPase activity. It revealed high confidence interactions with other subunits of DNA polymerase III (dnaE, dnaQ, holA) and proteins that have been described to be involved in replicative synthesis in bacteria. These interactions confirm the role of AUB74781.1 in DNA replication (Bargiela et al., 2020). The protein AUB74810.1 explained as pyrimidine/purine nucleoside phosphorylase showed strong interactions with proteins associated with the purine salvage pathway. However, the exact role of this protein in cold adaptation is unclear.

The protein AUB73565.1 which was found to contain a YceI-like domain showed high confidence interaction with Cytochrome b561. The gene that encodes cytochrome b561 is present upstream to yceI. Cytochrome b561 is an integral membrane protein involved in electron transfer activity. In some species, polyisoprenoid binding protein belonging to the YceI-like family was also found to co-exist with cytochrome b561. Thus, probably proteins with YceI-like domain are involved in electron transport system (Stancik et al., 2002; Handa et al., 2005). However, further analysis is needed to confirm its role in cold adaptation.

The gene encoding the protein AUB76544.1 demonstrated a proximal relationship with genes lpxA and lpxD. These genes were shown to be involved in the biosynthesis of lipid A, a component of LPS required for anchoring to the outer membrane of the cell (Kroeck et al., 2019). These functional interactions confirm the role of AUB76544.1 as a chaperone assisting in the proper folding and insertion of several outer membrane proteins. The protein AUB76897.1, containing Hsp70 like domain, exhibited a great degree of association with other chaperones like grpE, dnaJ, groEL. These proteins were closely related to promoting proper folding and assembly of unfolded polypeptides generated under stress conditions and were shown to have enhanced expression under cold stress (Chen et al., 2012). Hence it is evident that AUB76897.1 functions as a chaperone in maintaining the stability and function of stress-denatured proteins, crucial in cold conditions.

The protein AUB77315.1 was found to encompass the outer membrane porin (OprD) domain. From STRING analysis, the gene encoding OprD was found to be co-occurring with genes coding for porins, signifying a shared function (Szklarczyk et al., 2021). Though the confidence score of this interaction is less than our selected cut-off (>0.7), the results are consistent across the annotation pipeline. Hence, this protein can be inferred as an outer membrane porin involved in the transport across the membrane.

Proteins AUB73322.1, AUB74624.1, AUB75954.1, and AUB77446.1 were found to interact with proteins for which annotation is not available. For proteins AUB75187.1, and AUB75991.1, the confidence score of interactions is less than our selected cut-off (>0.7). Hence the interactions of these proteins were not considered for functional characterization. PPI networks are presented in Supplementary Figure S1 **.**

Finally, for each of the 25 HPs, we combined all the results from different annotation methods including domain analysis, gene ontology, sequence homology, sub-cellular localization, and PPIs to infer the final function. From our study, we could assign a function to 18 HPs with high confidence, of which 12 were found to be playing an important role in cold adaptation of *Pseudomonas* sp. Lz4W. The summary of this analysis is shown in Table 2. Complete results are provided in Supplementary Table S10.

**TABLE 2:** List of HPs and their inferred functions in relation to cold adaptation.

S.No	Protein ID	Inferred function	Role in cold adaptation
1	AUB73822.1	Peptidoglycan transglycosylase involved in cell wall biosynthesis	Cell wall biosynthesis
2	AUB74731.1	Thiosulfate sulfurtransferase	Not known
3	AUB74781.1	ATPase involved in DNA replication	DNA replication machinery
4	AUB74810.1	Pyrimidine/purine nucleoside phosphorylase involved in AMP biosynthesis	Not known
5	AUB77590.1	Glycosyltransferase	Not known
6	AUB75791.1	Phosphopantetheine transferase involved in post translation modifications of fatty acid synthases (FAS)	Fatty acid metabolism required for membrane fluidity
7	AUB75792.1	Phosphopantetheinyl transferase involved in post translation modifications of fatty acid synthases (FAS)	Fatty acid metabolism required for membrane fluidity
8	AUB76494.1	Flavinator of succinate dehydrogenase (SdhE)	Cellular respiration
9	AUB76830.1	Aminoglycoside phosphotransferase (APH)	Not known
10	AUB74223.1	OmpA family protein involved in electron transport	Energy metabolism
11	AUB76580.1	Outer membrane protein (OmpA) involved in respiratory chain	Energy metabolism
12	AUB76959.1	Outer membrane protein (OmpA) involved in respiratory chain	Energy metabolism
13	AUB73831.1	Lipopolysaccharide-assembly lipoprotein (LptE) involved In the assembly of lipopolysaccharide	Membrane structure and stability
14	AUB74026.1	Lipoprotein	Peptidoglycan biosynthesis
15	AUB75954.1	Copper resistance protein C involved in copper homeostasis	Not known
16	AUB76897.1	Molecular chaperone	Preventing the aggregation of stress-denatured proteins
17	AUB76544.1	Chaperone (skp)	Assembly and insertion of beta-barrel proteins in outer membrane
18	AUB73565.1	YceI family protein participating in ETC	Not known

## Conclusion

In general, HPs constitute about 30–50% of the microbial genomes, and the HP data is rapidly accumulating due to the deluge of sequencing data from sequencing technologies like NGS and mass spectrometry. Characterizing this HP data is one of the challenges in modern biomedical research. The genomic analysis of the Antarctic bacterium *Pseudomonas sp*. Lz4W revealed 743 CDS annotated as hypothetical proteins. Our proteomic analysis confirmed the expression of 61 hypothetical proteins at the translation level. This is the first study to characterize the HPs related to bacterial cold adaptation, revealing their role in membrane structure and stability, transport, protein folding, energy metabolism, and enzymatic regulation. Overall, our study once again highlighted the functional significance of hypothetical or uncharacterized sequences from the genomes. Furthermore, we have developed an integrated model based on mass spectrometry-based proteomics and systematic bioinformatics analysis to identify and characterize the uncharacterized proteins.

## Data Availability

The datasets presented in this study can be found in online repositories. The names of the repository/repositories and accession number(s) can be found in the article/ Supplementary Material- MS Proteomics data is available on PRIDE and can be accessed under the ID: PXD029741.
