# Supplementary material for: Investigating the Functional Role of Hypothetical Proteins From an Antarctic Bacterium Pseudomonas sp. Lz4W: Emphasis on Identifying Proteins Involved in Cold Adaptation
Source: Front Genet. 2022 Mar 11;13:825269. doi: 10.3389/fgene.2022.825269 (PMC8963723; doi:10.3389/fgene.2022.825269)
Supplement: Supplementary file 3 [file Table1.pdf]

Table S1. List of Bioinformatics tools used

Analysis	Bioinformatics tool	Version	URL
Functional annotation	CDD	3.19	https://www.ncbi.nlm.nih.gov/Structure/cdd/wrpsb.cgi
	SMART	9.0	http://smart.embl-heidelberg.de/
	CATH	4.3	http://www.cathdb.info/search/by_sequence
	Pfam	35.0	https://pfam.xfam.org/
	SUPERFAMILY	2.0	https://supfam.org/
	PROSITE	2021_04	https://prosite.expasy.org/
	InterPro	87.0	https://www.ebi.ac.uk/interpro/
	InterProScan	5.45-87.0	
Gene Ontology	GO FEAT	1.0	http://computationalbiology.ufpa.br/gofeat/
Subcellular localization	PSORTb	3.0.3	https://www.psort.org/psortb/
	CELLO	2.5	http://cello.life.nctu.edu.tw/
	SOSUI-GramN	*	https://harrier.nagahama-i-bio.ac.jp/sosui/sosuigramn/sosuigramn_submit.html
	SignalP	5.0	http://www.cbs.dtu.dk/services/SignalP/
	HMMTOP	2.0	http://www.enzim.hu/hmmtop/
	TMHMM	2.0	http://www.cbs.dtu.dk/services/TMHMM/
Physical-chemical characterization	ProtParam	*	https://web.expasy.org/protparam/
Protein-protein interaction network	STRING	11.5	https://string-db.org/
	Cytoscape	3.9.0	https://cytoscape.org/

*Information not available
