# Supplementary material for: Investigating the Functional Role of Hypothetical Proteins From an Antarctic Bacterium Pseudomonas sp. Lz4W: Emphasis on Identifying Proteins Involved in Cold Adaptation
Source: Front Genet. 2022 Mar 11;13:825269. doi: 10.3389/fgene.2022.825269 (PMC8963723; doi:10.3389/fgene.2022.825269)
Supplement: Supplementary file 6 [file DataSheet1.pdf]

Figure S1. Protein-protein interaction networks obtained from STRING analysis. Networks are visualized using Cytoscape (v 3.9.0).

- Networks are presented in circular layout with protein of study in centre.
- Edge connecting the protein of study and its important functional partners is highlighted in red.
- Width of the edge is proportional to the confidence score.
- Confidence score of the interaction is given in brackets.

A. Membrane proteins

1. AUB74223.1

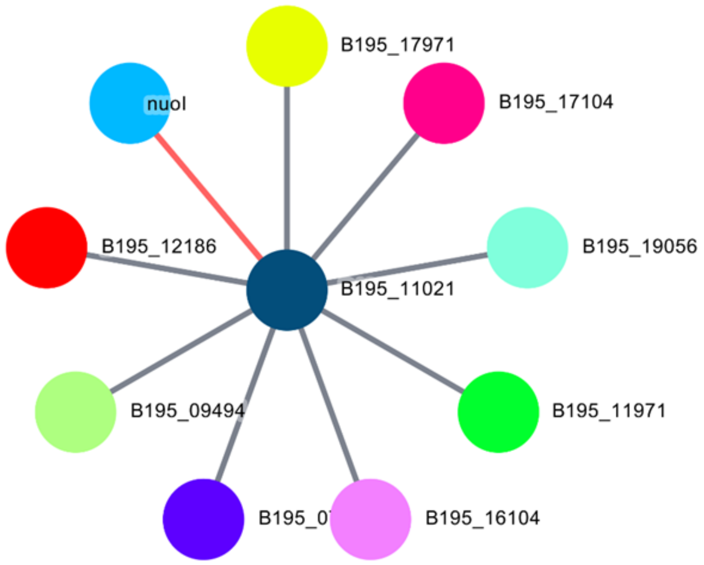

Functional domain: OmpA
Functional interactions:

- *nuoI* - NADH-quinone oxidoreductase subunit I (0.925)

Inferred function: Energy metabolism

2. AUB76580.1

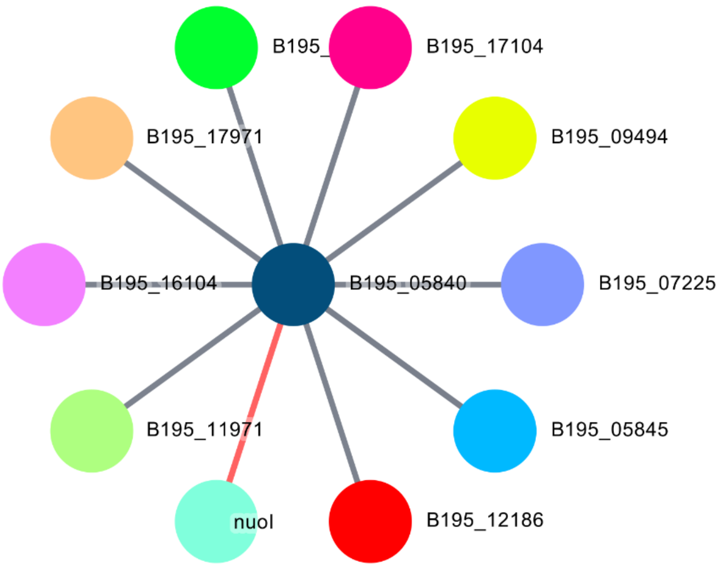

Functional domain: OmpA
Functional interactions:

- *nuoI* - NADH-quinone oxidoreductase subunit I (0.925)

Inferred function: Energy metabolism

3. AUB76959.1

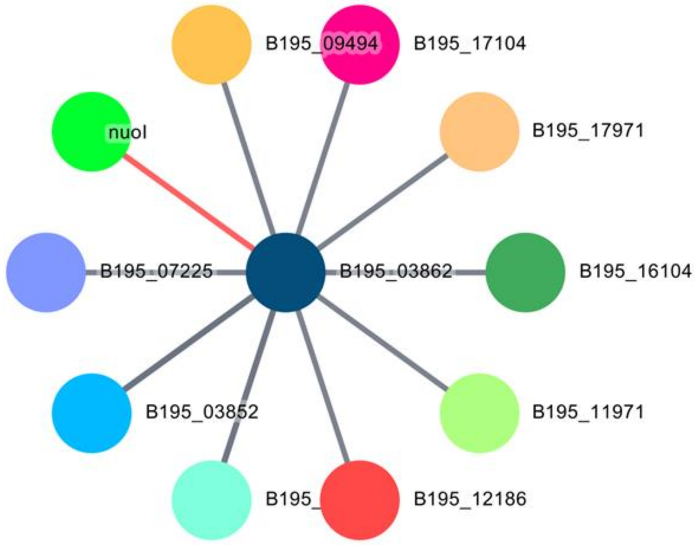

Functional domain: OmpA

Functional interactions:

- *nuoI* - NADH-quinone oxidoreductase subunit I (0.925)

Inferred function: Energy metabolism

B. Lipoproteins

4. AUB73831.1

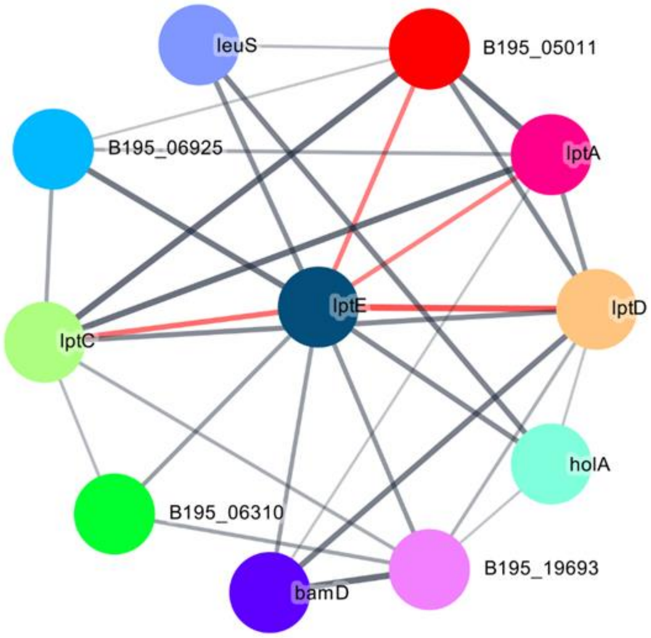

Functional domain: Lipopolysaccharide-assembly lipoprotein (LptE)

Functional interactions:

- lptD (0.999)
- lptC (0.851)
- B195_05011 (0.762)
- lptA (0.736)

Inferred function: Assembly of lipopolysaccharide

5. AUB74026.1

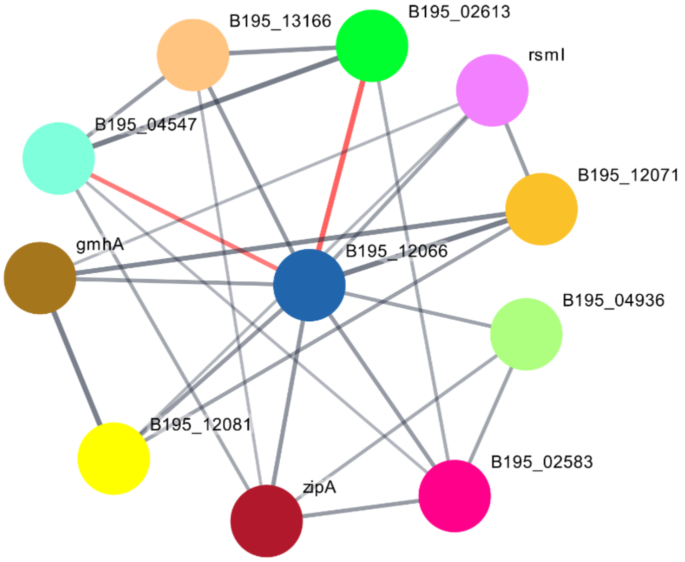

Functional domain: Lipoprotein
Functional interactions:

- B195_02613 - penicillin-binding protein 1A (0.929)
- B195_04547 - penicillin-binding protein 1B (0.783)

Inferred function: Peptidoglycan biosynthesis

C. Cellular Process

6. AUB73822.1

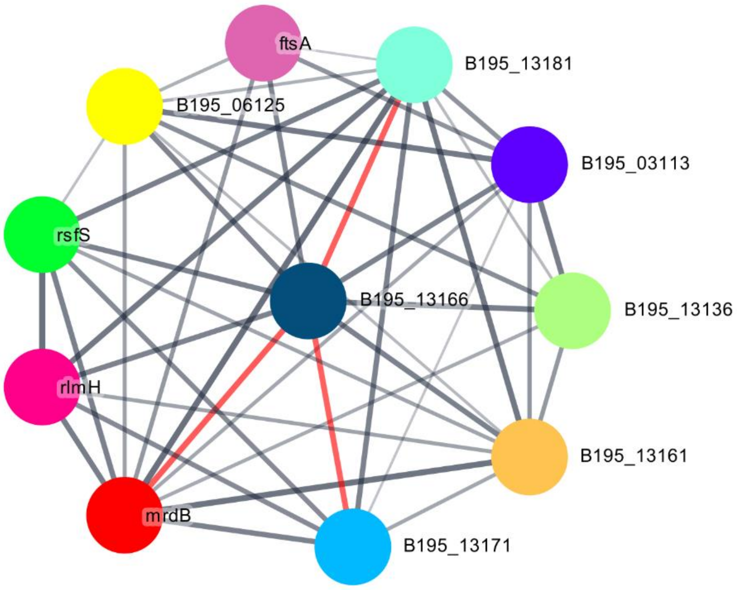

Functional domain: Endolytic peptidoglycan transglycosylase (RlpA)
Functional interactions:

- mrdB (0.965)
- mrdA (0.942)
- B195_13171 (0.936)

Inferred function: Cell wall biosynthesis

7. AUB75791.1

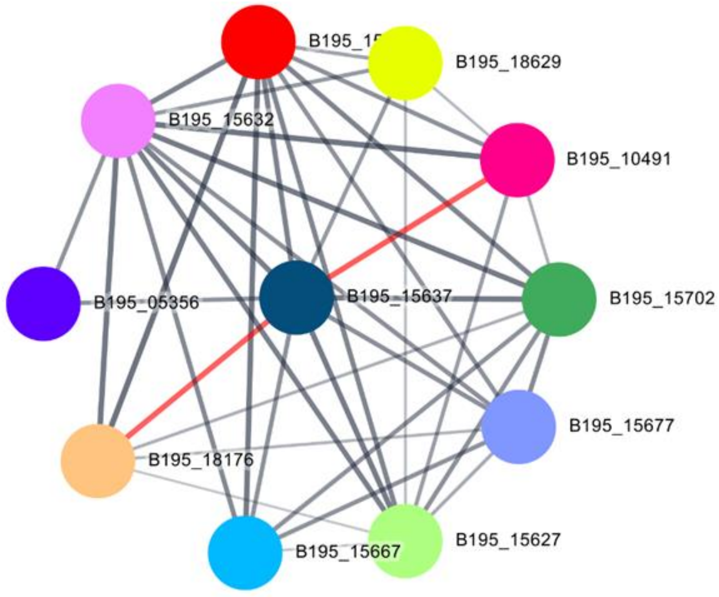

Functional domain: Condensation domain (Ligase)

Functional interactions:

- B195_10491 - 4'-phosphopantetheinyl transferase (0.955)
- B195_18176 - Beta-ketoacyl synthase-like protein (0.936)

Inferred function: Fatty acid biosynthesis

8. AUB75792.1

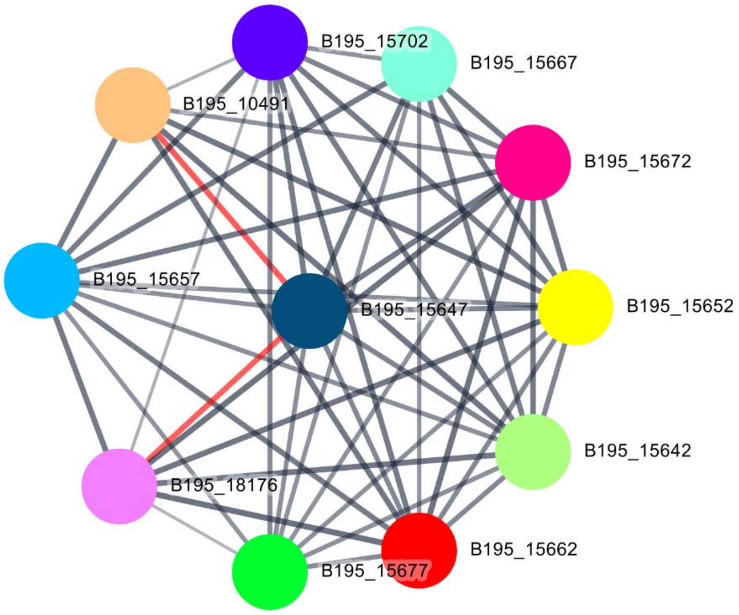

Functional domain: Condensation domain (Ligase)

Functional interactions:

- B195_10491 - 4'-phosphopantetheinyl transferase (0.955)
- B195_18176 - Beta-ketoacyl synthase-like protein (0.936)

Inferred function: Fatty acid biosynthesis

9. AUB76494.1

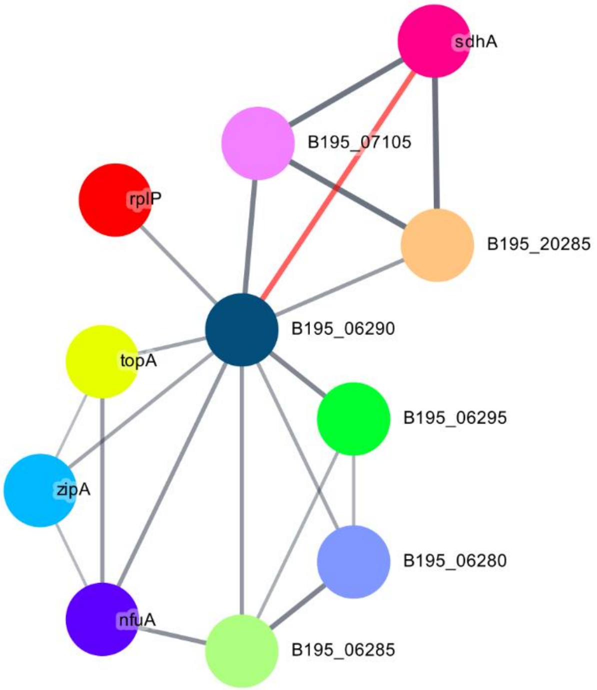

Functional domain: Flavinator of succinate dehydrogenase

Functional interactions:

- sdhA (0.937)

Inferred function: Cellular respiration

10. AUB74781.1

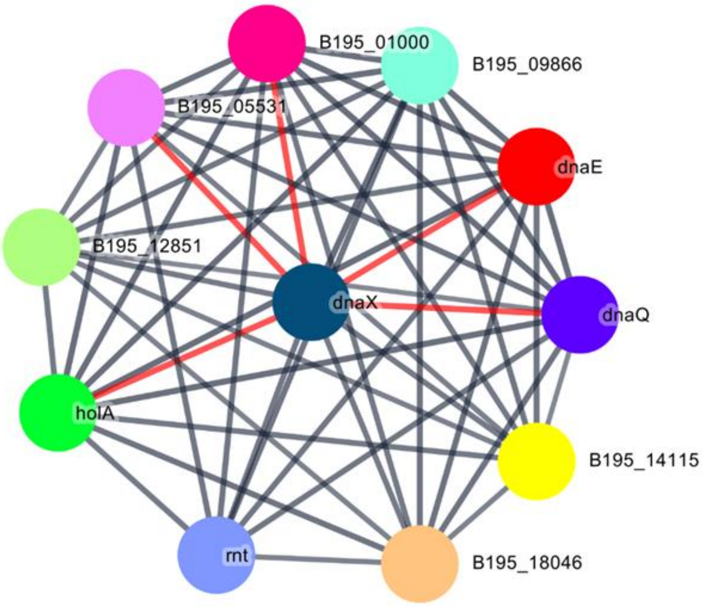

Functional domain: ATPase domain (DNA polymerase III subunit)

Functional interactions:

- B195_01000 - DNA tethering and processivity (0.999)
- B195_05531 - chi subunit (0.999)
- dnaE (0.999)
- dnaQ (0.999)
- holA (0.999)

Inferred function: DNA replication

D. Chaperones

11. AUB76544.1

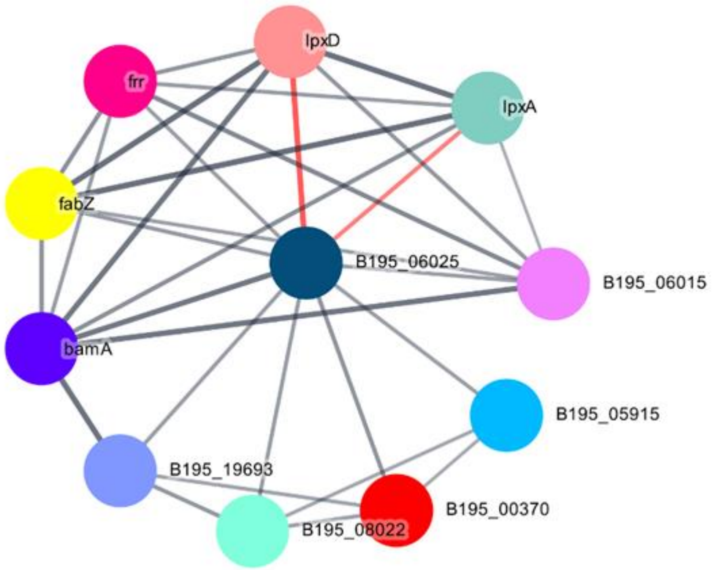

Functional domain: Outer membrane chaperone (skp)
Functional interactions:

- lpxD - Biosynthesis of lipid A (0.970)
- lpxA - Biosynthesis of lipid A (0.746)

Inferred function: Folding and insertion of several outer membrane proteins

12. AUB76897.1

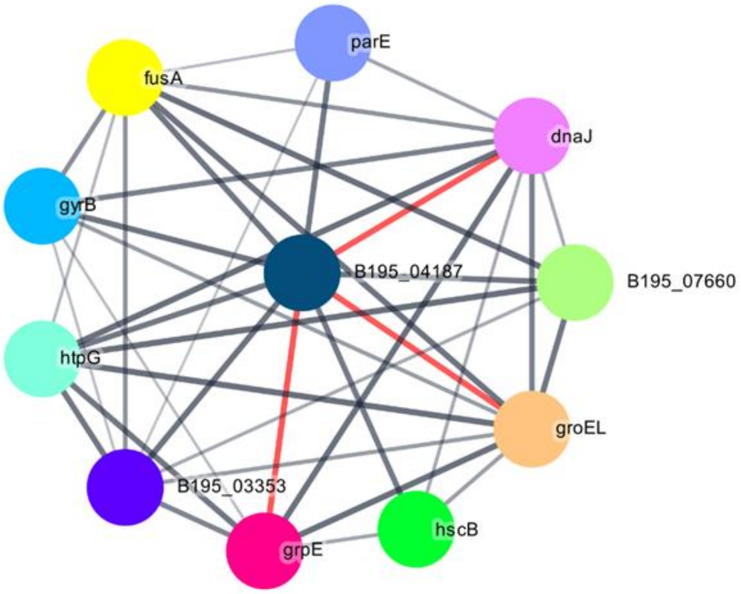

Functional domain: Hsp70 like domain
Functional interactions:

- grpE (0.990)
- dnaJ (0.984)
- groEL (0.977)

Inferred function: Molecular chaperone maintaining the stability and function of stress-denatured proteins

AUB77315.1*

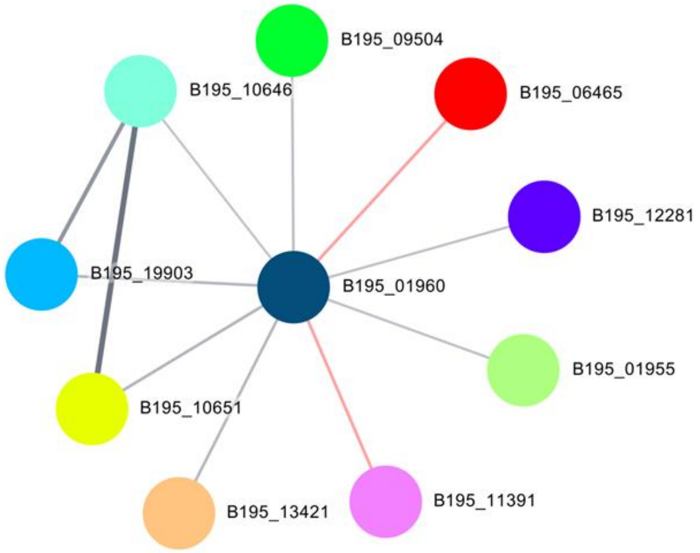

Functional domain: OprD

Functional interactions:

- B195_06465 - Porin (0.544)
- B195_11391 - Porin (0.537)

Inferred function: Outer membrane porin

***** Confidence score of the interactions is less than the selected cut-off**.**
